# Recommendation Paper on Advancing the Use of Decentralised Elements in Clinical Trials

**DOI:** 10.1007/s43441-025-00796-w

**Published:** 2025-05-27

**Authors:** Alison Bond, Tracey Robertson, Christine Fletcher, Elizabeth Theogaraj, Greg Jordinson, Scott Askin

**Affiliations:** 1 Director, Global Regulatory Policy and Intelligence, Amgen Ltd, 4 Uxbridge Business Park, Sanderson Road, Uxbridge, UB8 1DH United Kingdom; 2 Boehringer Ingelheim Pharmaceuticals Inc., Ridgefield, Connecticut USA; 3 GSK, Stevenage, United Kingdom; 4 Roche, Basel, Switzerland; 5 Johnson & Johnson, High Wycombe, United Kingdom; 6 Novartis, Basel, Switzerland

**Keywords:** Clinical trials, Decentralised clinical trials, Access, Innovation, Rare diseases

## Abstract

The expansion of the use of decentralised elements in clinical trials in the European Union is driven by the opportunities they offer, including their potential to enhance trial access and improve research outcomes. Despite these potential benefits, several regulatory, operational, and technological challenges impede their widespread adoption. This paper, developed by members of the European Federation of Pharmaceutical Industries and Associations, proposes strategies and policy recommendations to overcome these barriers. Through harmonised regulatory frameworks, improved data validation methods, and increased stakeholder collaboration, the use of decentralised elements could become a standard practice in clinical research as part of the clinical trial toolbox to be deployed as appropriate, thereby enabling innovation and allowing for equitable access to clinical trials for diverse populations.

## Introduction

This paper discusses the industry’s perspective and experience regarding the use of decentralised elements in clinical trials (for simplification, also referred to as Decentralised Clinical Trial (DCT) elements) in the European Union (EU). The objective is to support the use of DCT elements where appropriate, as standard practice in clinical trials to realise and maximise the perceived and experienced benefits for trial participants and research. This paper also proposes recommendations to overcome the perceived and identified challenges regarding the adoption of DCT elements in clinical trials in the EU. This paper argues that DCT elements in clinical trials offer valuable opportunities for participants and researchers and that the EU and global regulatory framework needs to evolve further to support the uptake and development of such practices.

This paper stems from an initiative spearheaded by members of the European Federation of Pharmaceutical Industries and Associations (EFPIA), a trade association representing the pharmaceutical industry operating in Europe. The views presented in this paper arise from various collaborative initiatives that have involved various stakeholder groups and organisations around the world. These include trade associations, Public–Private Partnerships, industry consortia, and regulator-led initiatives, with the same aim of advancing the optimal use of DCT elements to optimise drug development and benefit all involved in drug development including trial participants.

While the use of DCT elements in clinical studies is not new, the decoupling of site and study during clinical trials was accelerated during the COVID-19 pandemic. The uptake of DCT elements enabled trial continuity during the pandemic. As travelling restrictions applied, novel ways of conducting clinical trials were developed to continue trials [1] during these unprecedented times. Greater utilisation of new technologies, such as wearables and telehealth consultations, as well as the increased use of facilities local to the participants, became central in clinical studies during the pandemic. While traditional site-based clinical trials faced difficulties in reaching their pre-pandemic results, decentralised trials recovered faster from the impact of COVID-19 in terms of participant enrolment and discontinuation rates [2].

Clinical trials with DCT elements offer unprecedented opportunities to improve access for participants for whom access to traditional fully site-based clinical trials may prove challenging. By designing the clinical trials around participants as opposed to research facilities, trials incorporating DCT elements may benefit from increased participant retention rates [3] and widen the participant pool to those normally excluded by geographical restrictions. Furthermore, the use of DCT elements may enable innovation in clinical trials more broadly, such as facilitating the use of pragmatic trials [4]. The use of DCT elements in clinical trials is poised to particularly benefit research on paediatric, chronic, and rare diseases [5], echoing EU priorities. Rare diseases have been one of the EU’s priorities for the last two decades [6] and research on medicines for paediatric use remains insufficient, as identified by the European Commission (EC) [7].

In turn, this benefits scientific research, contributing to the EU’s scientific reputation and attractiveness in line with the Accelerating Clinical Trials in the EU (ACT EU) initiative, co-led by the EC, European Medicines Agency (EMA) and the Heads of Medicines Agencies (HMA) [8]. Scientific excellence significantly bolsters the EU's competitiveness and strategic autonomy by driving innovation through the development of cutting-edge solutions that address societal challenges, thereby strengthening the EU's global leadership. Furthermore, this contributes to reducing reliance on external sources for research, ensuring the EU’s self-reliance to the benefit of its citizens and contributing to positioning the EU as a global leader in research.

This paper presents the industry’s experience and positions regarding the use of DCT elements in clinical trials. The views and recommendations expressed are based on discussions of experience and shared insights from EFPIA members as part of the EFPIA DCT Team, which was formed in September 2021. These insights were used to develop positions and recommendations, some of which have been discussed and developed at multi-stakeholder workshops and conferences, and shaped EFPIA input and comments on documents related to the use of DCT elements in clinical trials.

To gain further insight into the industry's experience with deploying DCT elements in clinical trials, a survey of EFPIA member company experience was conducted between October 2023 and March 2024. The survey was distributed to EFPIA member companies and addressed the following key areas:

1. Why a DCT element was selected and the anticipated benefits, and whether those benefits were realised or not, along with the reasons for anticipated benefits not being realised.
2. Challenges faced with implementation of the DCT element, and how those challenges were overcome.
3. The submission of data from clinical trials utilising DCT elements for regulatory applications, and the feedback received regarding the acceptability of the generated data.

Nine companies responded to the survey, providing some valuable insights into the first two areas regarding the benefits and challenges with adoption of DCT elements in clinical trials across various therapeutic areas including Oncology, Dermatology, Haematology, Cardiovascular, and Neurology. Minimal information was received in response to point 3 regarding the submission of data from clinical trials utilising DCT elements, because the trials had not yet been completed or the data had not yet been reviewed by regulators at the time the survey was conducted.

The paper first outlines the opportunities of utilising DCT elements in clinical trials, then discusses the challenges impeding the uptake of DCT elements in the EU. Finally, this paper presents recommendations, developed in collaboration with relevant stakeholders and based on the collected insights and industry experience, to mitigate the societal, regulatory and legislative hurdles impeding the wider use and recognition of DCT elements as valuable contributions to participants and research.

## Overview and Context: Decentralised Elements in Clinical Trials

Clinical trials with decentralised elements became increasingly used in the context of the COVID-19 pandemic. This played a central role in accelerating the development and uptake of decentralised practices. In particular, the advancement of telemedicine facilitated virtual assessments to be conducted [9] when participants were not allowed to travel to the research facilities during the pandemic. In turn, this contributed, in the short-term at least, to a wider acceptance of such practices, impacting the design of clinical trials and making them less dependent on the research facility conducting the trial.

The key difference between “traditional” fully site-based clinical trials and studies with DCT elements is the attachment to the research site. While in the former all clinical trial activities happen in the main site, the latter model encompasses uniquely designed trials that combine one or more decentralised elements. The selection of deployed DCT elements depends on the objectives and conditions of the clinical trials, the safety/administration profile of the Investigational Medicinal Product (IMP), the participant population and needs, and the local regulatory framework.

Decentralised elements include telemedicine; mobile healthcare providers (e.g., home nurses, mobile clinics); local healthcare providers (e.g., general practitioners); local laboratories and imaging centres; direct to participant IMP shipment; electronic informed consent or remote consenting; concierge services; digital health technologies (DHTs) (e.g., wearables or apps); recruitment through digital channels (Digital Recruitment e.g. social media campaigns, online pre-screeners, trial websites). Some additional DCT elements are already widely used, especially e-PRO/e-COA (electronic Patient Reported Outcomes/electronic Clinical Outcome Assessment), and their use is already well-established in site-based “traditional” trials. This definition of decentralised elements builds upon the definitional language from the Clinical Trials Transformation Initiative (CTTI).

## Industry-Identified Opportunities: Bringing Clinical Trials Closer to Participants and Improving Research

The use of decentralised elements embodies the convergence of interests between participants and trial results. As with traditional site-based clinical trials, this approach prioritises participants’ rights, safety, and well-being while ensuring data integrity. There is the potential for significant mutual benefit of this approach, when employed appropriately to clinical trials.

### Improving Access to Clinical Trials

Clinical trials with decentralised elements present significant opportunities when it comes to access**.** They enable easier access to clinical development and therapies and allow flexibility for the trial to better fit around the participants’ daily lives.

DCT elements help bridge the geographical restrictions of site-based trials. The dependence on a particular site is associated with disruption to family life and work commitments, as well as heightened travel costs, further increasing the burden of the ailment on participants. DCT elements in clinical trials thus may make clinical trials less disruptive for participants, offering the potential of increasing participant retention and decreasing missed visits.

### Surveyed Industry Experience: The Anticipated Benefits of Direct-to-Participant IMP Shipment

Several approaches enable bringing the clinical trial to the participant, rather than the other way round. Direct-to-Participant (DtP) IMP shipment is one of these approaches, allowing the shipment of IMPs to the participant’s home. The anticipated benefit was increasing patient convenience linked to fewer site visits, thus limiting the disruptive effect of the clinical study on participants’ daily lives. In the EU, national regulators are generally not in favour of delivery of the IMP to the trial participant’s home from a depot (as opposed to from the site) due to concerns over delivery, storage, data privacy as well as treatment compliance. The HMA, EC, EMA recommendation paper on decentralised elements in clinical trials, however, addresses these concerns by outlining best practices enabling the full benefits of DtP delivery methods. DtP delivery has been implemented in conjunction with home nurse visits and telemedicine to decrease travel burden and potentially enhance participant retention (although not yet demonstrated at the time of industry experience collection). Detailed training for sites played a crucial role in ensuring proper adherence to the process. Due to varying national provisions, DtP delivery methods should be considered carefully during clinical trial planning.

Furthermore, some communities may be underserved due to a lack of proximity to trial sites, including participants living in rural or remote areas. DCT elements promise to reduce this obstacle by limiting the occurrence of participants’ on-site visits, allowing geographically dispersed patients to partake in studies [10].

This opportunity of improved clinical trial access is particularly relevant to the study of rare diseases. As the name suggests, rare diseases are of low prevalence, affecting a small portion of the population, in the EU no more than 1 person in 2000 [11], making it difficult to recruit sufficient participants in a given region. By increasing access to clinical trials, in turn, this may improve participant diversity, contributing to improving the scientific understanding of studied ailments. Those suffering from rare diseases for which data is lacking, are particularly poised to benefit from this approach. The use of decentralised elements is also particularly beneficial for paediatric indications, and for people for whom travelling to the site may be especially difficult e.g., some elderly or less mobile participants. The decentralised nature of DCTs thus brings them closer to participants, contributing to reducing healthcare access gaps [12]. This also aligns with the EU’s attempt to reduce health inequalities [13].

### Surveyed Industry Experience: The Anticipated and Realised Benefits of Telemedicine

The industry experience survey demonstrated attempts to deploy telemedicine with a view to participant and site convenience, complement home nursing, reduce travel burden and expenses, improving participation and retention of participants and reducing missed visits. Responses to the industry experience survey indicated that participant retention was improved and missed visits were reduced in number, however there were challenges, largely due to technical issues and regulatory hurdles, discussed later in the paper.

### Scientific Benefits of Using Decentralised Elements

Clinical trials with DCT elements must be performed to the same standards as traditional site-based trials, responding to the highest standards of scientific research, thus contributing to knowledge building and clinical breakthroughs. The use of DCT elements may enable diversity as well as continuity of data collection, leading to more reliable and generalisable results over time.

Data collected using DCT elements must be scientifically sound and not compromised by the remote assessments, especially when gathered via digital tools that require validation. The risk of questioning of the data generated by such clinical trials can make sponsors reluctant to run such trials with DCT elements. However, reported practices attest to the systematic use of the V3 validation framework to assess the reliability and accuracy of the collected data for digital/DHTs [14]. This involves three levels of validation evidence: verification, analytical validation, and clinical validation. Clinical trials with DCT elements thus contribute in their own right to scientific knowledge.

DCTs may enhance the objectivity, sensitivity, and quality of clinical assessments through the use of expert remote rating via telemedicine or central rating of imaging endpoints. Remote or virtual assessments have been deemed more objective than in-person assessments. The use of DHTs, especially wearables and apps allow remote and novel measures, collected in real-time and potentially continuously outside of the research site, offering opportunities for more complete and meaningful data. While remote assessments can enhance objectivity in certain ways, they also introduce new variables that can affect fairness and accuracy. The effectiveness often depends on the design of the assessment and the measures taken to mitigate potential biases.

### Surveyed Industry Experience: The Anticipated and Realised Benefits of Digital Data Collection

The anticipated benefits of digital data collection were enhanced compliance, real time data collection to help sites understand participant experience, enhanced data quality, patient convenience, faster recruitment, and increased retention. The realised benefits reported by some survey respondents were participant convenience, collection of data through an App and the patients were satisfied with the simplicity of eDiaries, and digital data collection allowed for oversight, monitoring and reconciliation of eCOA data.

## Industry-Identified Challenges

As elaborated in the previous section, the use of decentralised elements in clinical trials represents a promising evolution in clinical research, providing opportunities for increased participant engagement, improved access and sound scientific research. However, several challenges must be addressed to realise their full potential.

One of the primary challenges is the complexity of data collection, security and management. Clinical trials with DCT elements rely heavily on digital health technologies, such as wearables and mobile apps, to collect data remotely. Ensuring the integrity, consistency, and security of this data across various sources is crucial. This complexity can lead to selection bias if technological access is not uniform among all participants, potentially skewing the study results towards more tech-savvy individuals​.

Another significant challenge lies in ensuring clear roles and oversight within trials with decentralised elements. The decentralised nature of these trials can complicate relationships between investigators, sponsors, and third-party providers. Maintaining robust communication and clearly defined roles is essential to uphold trial integrity and participant safety. Resistance to new technologies and processes from participants and caregivers can also hinder the implementation of decentralised elements, potentially affecting data quality and overall trial outcomes​. In addition, further assessment is needed to understand site challenges with the use of decentralised elements in clinical trials, in order to develop strategies to work together to overcome these, considering also the work of TransCelerate [15], Trials@Home [16] and the Society for Clinical Research Sites [17].

### Surveyed Industry Experience: Participant Push-Back on DCT Elements

Home health visits were deployed by sponsors with the anticipated benefit of lowering participant burden and improving recruitment, with improved convenience for elderly participants with limited mobility and/ or significant co-morbidities Some survey respondents reported that some participants were more comfortable with on-site visits, either resulting from their condition, or simply due to personal or generational preferences.

Data privacy and protection remain significant concerns, especially with the reliance on remote data collection tools. Compliance with global and local regulations, such as the General Data Protection Regulation (GDPR) in the European Union, presents a significant challenge that requires diligent planning and execution. Additionally, ensuring the comparability of remotely collected data with on-site data is crucial to meet regulatory requirements. This demands rigorous validation and quality assurance processes to ensure data reliability​.

Technical challenges in remote assessments can also be problematic, particularly when working with third-party vendor systems, and sponsors may need to step in and provide assistance such as rapid response helpdesk teams. Mitigations may need to be put in place to address concerns over participants' contact details, or if apps cease working, which could potentially affect the collection of data. Operational complexity can be mitigated by e.g. using specialised DCT vendors offering several DCT elements under one Login access, good site helpdesk, and appropriate site training. Also, engagement with the sites is a key factor for site acceptance of the proposed DCT elements. i.e. already at the feasibility level. To ensure the feasibility and user-friendliness of DCTs, the management of these elements ought to be as simple as possible.

### Surveyed Industry Experience: The Challenges with Digital Data Collection

Whilst data collection via an app also enabled monitoring and reconciliation of e-COA data, some survey respondents also reported significant costs and resource investment associated with vendors and site use of systems. Trial participants also needed support for tool familiarisation (it is part of the investigator’s responsibilities to ensure participants are comfortable in using these tools). Technical issues were overcome with additional support such as rapid response helpdesk teams. Some survey respondents also noted language barriers held back deployment. Whilst regulators are becoming more familiar with this approach, sponsors must ensure compliance with diverse privacy laws in the EU. Some Sponsors withdrew this element due to implementation difficulties. One survey respondent additionally noted the necessity of validating the identity of the individual inputting data.

Finally, the regulatory landscape for the use of DCT elements in clinical trials is highly fragmented, with varying national requirements posing significant challenge. The lack of harmonised guidelines can lead to uncertainty and increased complexity in trial design and execution. Efforts to align regulatory frameworks globally, such as through initiatives like the International Council for Harmonisation (ICH) E6 (R3) guideline, are essential to streamline the implementation of DCTs and ensure their broader adoption. Alignment across the EU Member states is also needed, with the varying requirements highlighted in the National Provisions Overview of the EU recommendation paper on decentralised elements in clinical trials [18]. While a clear and centralised regulatory framework remains absent in the EU, the National Provisions Overview [18] is helpful for navigating across Member States.

### Surveyed Industry Experience: Examples of Fragmented EU Legislation – Telemedicine, eConsent, DtP IMP, Digital Recruitment

As discussed previously, the industry experience survey indicated anticipated and realised benefits of **telemedicine**, however there were challenges. Regarding regulatory concerns, within the EU the disparity between countries means sponsors must consider national laws and regulations that restrict the use of telemedicine e.g. within Germany. For **e-Consent**, variability between EU countries was also reported by some respondents to the industry experience survey. In some countries, such as Sweden, video was used during remote eConsent. Some require careful management to ensure privacy (e.g. avoiding the use of participants email addresses in France) and Denmark allows eConsent if there is active dialogue with the participant – the use of pre-recorded video was not acceptable. Among the industry experience survey responses received, some respondents stated that they removed this DCT element due to the challenges experienced in operationalising this element. As mentioned previously, due to varying national provisions, **DtP IMP** delivery methods should be considered carefully during clinical trial planning. For **Digital Recruitment,** the anticipated benefits ranged from demonstrating feasibility to faster recruitment and the potential to reach large numbers of patients quickly. However, survey respondents reported challenges e.g., issues regarding privacy protection, high screening failure rates and inexperienced vendors. Barring vendor issues, Digital Recruitment deployment in the US was seen as simpler than in EU where country tailored approaches were needed.

See Table 1 for examples of issues/gaps in regulatory clarity and variability for DCT elements, as reported in the industry experience survey.

**Table 1:** Examples of Issues/gaps in Regulatory Clarity and Variability for DCT Elements, as Reported by Respondents to the EFPIA Industry Experience Survey.

DCT Element	Issues/ gaps in regulatory clarity and variability
Telemedicine	National laws and regulations may restrict the use of telemedicine in Germany, where specific technical requirements must be met according to local requirements
eConsent	Variability between EU countries was reported by some respondents to the industry experience survey. In some countries, such as Sweden, it was reported that video was required during remote eConsent. Some require careful management to ensure privacy (avoiding the use of participants email addresses in France) and Denmark allows eConsent if there is active dialogue with the participant – the use of pre-recorded video was not acceptable. Some survey respondents stated that they removed this DCT element due to the challenges experienced in operationalising this element
DtP IMP	Due to varying national provisions, DtP IMP delivery methods should be considered carefully during clinical trial planning. In the EU, national regulators are generally not in favour of delivery of the IMP to the trial participant’s home from a depot (as opposed to from the site) due to concerns over delivery, storage, data privacy as well as treatment compliance
Digital Recruitment	The industry experience survey reported issues regarding privacy protection. Digital Recruitment deployment in the US was seen as simpler than in the EU where country tailored approaches were needed

### Sponsor Considerations When Evaluating Deployment of DCT Elements

Decisions on the use of DCT elements often overlook operational complexities, focusing mainly on scientific perspectives or patient-centricity. Optional DCT elements while preferable, may increase complexity, complicating sponsor oversight and requiring more effort in managing data flow, integration, and reconciliation. Global studies struggle with timely information on permissible DCT elements across jurisdictions and sponsors must ensure compliance with all appropriate data privacy regulations for digital DCT elements and address these in the Informed Consent Form (ICF). In addition, it is generally not possible to contract with global vendors to provide local DCT services in all countries.

Sponsors need to consider and address these challenges when implementing DCT elements in clinical trials. The use of DCT elements should be tailored to the individual trial with trial-specific rationale. DCT elements, if used, should be selected to fit the scientific question, IMP, trial design and the participant population. Consideration of local requirements in each country in which the trial is run is also needed. Engagement of patients, advocates and Health Care Professionals (HCPs) in trial design, including DCT elements, is highly recommended and may help address participant hesitance with the use of some DCT elements. Hybrid models with choice and flexibility are also recommended where possible, with consideration given to the complexity that this may incur. Working through initiatives such as CTTI, TransCelerate and Innovative Health Initiative (IHI), and sharing experiences and learnings, sponsors and other stakeholders are working towards a shared understanding and ways to address the challenges.

Key to moving forward is sharing of experience and learnings between stakeholders. This is difficult until more examples of clinical trials with DCT elements are submitted to regulators for review. It is imperative to learn from key initiatives and studies, such as best practices from IHI Trials@Home. Trials@Home [16] is aimed at exploring the potential of digital technologies for use in decentralised clinical trials. The project, through the RADIAL trial [19], will compare, among other parameters, the patient satisfaction and data quality generated by 1. A traditional clinical trial setting, 2. A completely decentralised clinical study, and 3. A hybrid approach comprised of on-site and remote activities. Aimed at gathering concrete knowledge on the different approaches, this initiative promises to outline best practices, enabling clinical trial stakeholders, including industry and regulators, to drive the continuous improvement of DCT methodologies​. Sponsors are also actively engaging with regulators and other stakeholders at multi-stakeholder workshops and conferences, as well as commenting on draft guidance to shape the future regulatory environment to enable and advance the use of DCT elements in clinical trials.

## Industry Recommendations

To fully harness the potential of DCT elements in clinical trials, and facilitate their implementation and acceptance, it is essential to address several critical needs. Chief among these is the harmonisation and alignment of regulatory frameworks, both within the EU and globally. The following section outlines key recommendations addressed to EU and global regulators, and other stakeholders including industry, in the hope of enabling and streamlining the use of DCT elements to the benefit of participants and scientific research.

### Recommendation 1: Create a Harmonised Regulatory Framework in the EU

Within the EU, the regulatory landscape for DCT elements is currently fragmented, with varying requirements across Member States. This lack of consistency can complicate the design and execution of trials, as sponsors must navigate disparate national regulations. A unified approach is necessary to streamline processes and ensure that clinical trials with DCT elements are conducted efficiently across borders. Harmonising or aligning EU regulations would help facilitate access to clinical trials, allowing for a more diverse and representative participant pool. This would involve working with National Competent Authorities and National Trade Associations to see where alignment can be sought.

### Recommendation 2: Develop Globally Aligned Guidelines

On a global scale, the development of harmonised guidelines such as ICH E6 (R3) [20] is crucial. This guidance may help towards providing a framework or standards for the implementation of DCT elements, ensuring that trials are conducted in a standardised manner worldwide and in line with Good Clinical Practices. Stakeholder commenting to shape draft guidance from different regulators and regions may also help to align perspectives and promote consistency. Global alignment will not only reduce the complexity of conducting international trials but also foster greater collaboration and sharing of data across regions, ultimately benefiting participants and advancing scientific knowledge.

### Recommendation 3: Establish Clear Criteria for Evaluating the Collected Data

This scientific community should establish clear criteria for evaluating data collected through decentralised methods to ensure it meets the same rigorous standards and is comparable to traditional trials. This includes validating the reliability and accuracy of data collected via digital health technologies and remote monitoring, as suggested in the V3 validation framework for digital/DHT. Recognising the validity of DCT data will encourage sponsors to integrate decentralised elements into their trials, fostering innovation and improving outcomes​.

### Recommendation 4: Support the Sharing of Best Practices and Lessons-Learned

As the ecosystem gains more experience with DCT elements, it is essential to share expertise and best practices among stakeholders for change management. This could include documenting successful strategies and lessons learned from past trials to guide future efforts. Transparency from regulators on their review and assessment of DCT elements and data generated using them could also be considered. Sharing knowledge will help to standardise processes, reduce redundancies, and accelerate the implementation of DCT elements across various therapeutic areas. Collaboration among trade associations, industry stakeholders, and regulatory bodies, as well as at the site and participant level, is key to developing a shared understanding of DCTs. Additionally, public–private partnerships ought to be supported, and learnings integrated into practice.

## Conclusion

Decentralised elements in clinical trials offer opportunities beneficial for both participants and scientific research, and there has been activity by various stakeholders, including industry and regulators, to enable their use over several years. The COVID-19 pandemic served to highlight the importance and value of decentralised elements and catalysed their implementation. The increased accessibility of clinical studies across geography and the continuity of data collection leading to more reliable and valid results over time, are among the clear advantages of this approach.

To enable broader implementation of DCT elements in clinical trials, the systematic use of such methods must be supported by evolution in the regulatory environment and based on best practices. Important strides on this matter have been made in recent years in the EU, notably through the work of EMA, HMA and EC, with the recommendation paper on decentralised elements in clinical trials. Additionally, the organisation of multi-stakeholder workshops, conferences and initiatives demonstrate clear willingness to support the uptake of DCT elements.

However, as the industry experience has indicated, several challenges stand in the way of full deployment of DCT elements as common practice. To overcome these obstacles, the regulatory framework, both in the EU and globally, needs to be aligned to allow utilisation of DCT elements. A collaborative effort with all the key stakeholders is needed to share, and build upon, experience (including best practices) to overcome the identified challenges. Industry remains dedicated to the use of DCT elements in clinical trials and is eager to collaborate with regulators, researchers, sites, clinical trial participants and other relevant stakeholders to take forward the recommendations set out in this paper, to advance the adoption of DCT elements in clinical trials.

Finally, it must be reiterated that clinical trials with decentralised elements are not meant to replace “traditional” site-based clinical trials, but rather to complement them, as a further asset in the clinical trial toolbox. The foreseen benefits offer innovation benefiting European patients and heightened scientific excellence, contributing to the attractiveness and competitiveness of the EU healthcare industry on the global stage.

## Data Availability

No datasets were generated or analysed during the current study.

## References

[1] Suman A, van Es J, Gardarsdottir H, et al. A cross-sectional survey on the early impact of COVID-19 on the uptake of decentralised trial methods in the conduct of clinical trials. Trials. 2022. 10.1186/s13063-022-06706-x. 36203202 10.1186/s13063-022-06706-x PMC9535935

[2] Price J, Goodson N, Warren EJ, et al. Resilient design: decentralized trials recovered faster from the impact of COVID-19 than traditional site-based designs. Expert Rev Med Devices. 2021;18(sup1):1–4. 10.1080/17434440.2021.2014818. 34894989 10.1080/17434440.2021.2014818

[3] Miyata BL, Tafuto B, Jose N. Methods and perceptions of success for patient recruitment in decentralized clinical studies. J Clin Transl Sci. 2023;7(1):e232. 10.1017/cts.2023.643. 38028356 10.1017/cts.2023.643 PMC10643920

[4] Clinical Research Expert Group (CREG) and Integrated Evidence Generation and Use (IEGU) Working Groups. An EFPIA position paper on randomised pragmatic trials to generate high-quality real-world evidence for regulatory decisions. 2023 June 21. https://www.efpia.eu/media/guokpw0b/an-efpia-position-paper-on-randomised-pragmatic-trials-to-generate-high-quality-real-world-evidence-for-regulatory-decisions.pdf

[5] de Jong AJ, van Rijssel TI, Zuidgeest MGP, et al. Opportunities and challenges for decentralized clinical trials: european regulators’ perspective. Clin Pharmacol Ther. 2022;112(2):344–52. 10.1002/cpt.2628. 35488483 10.1002/cpt.2628 PMC9540149

[6] European Commission. Rare diseases. Public Health. 2023. https://health.ec.europa.eu/rare-diseases-and-european-reference-networks/rare-diseases_en

[7] European Commission.. Medicines for children. Public Health. 2024. https://health.ec.europa.eu/medicinal-products/medicines-children_en

[8] European Medicines Agency. Accelerating Clinical Trials in the EU (ACT EU). (n.d.). https://www.ema.europa.eu/en/human-regulatory-overview/research-development/clinical-trials-human-medicines/accelerating-clinical-trials-eu-act-eu

[9] Van Norman GA. Decentralized clinical trials: the future of medical product development? JACC Basic Transl Sci. 2021;6(4):384–7. 10.1016/j.jacbts.2021.01.011. 33997523 10.1016/j.jacbts.2021.01.011 PMC8093545

[10] Ghadessi M, Di J, Wang C, et al. Decentralized clinical trials and rare diseases: a drug information association innovative design scientific working group (DIA-IDSWG) perspective. Orph J Rare Dis. 2023. 10.1186/s13023-023-02693-7. 10.1186/s13023-023-02693-7 PMC10088572 37041605

[11] REGULATION (EC) No 141/2000 OF THE EUROPEAN PARLIAMENT AND OF THE COUNCIL of 16 December 1999 on orphan medicinal products. https://eur-lex.europa.eu/legal-content/EN/TXT/PDF/?uri=CELEX:32000R0141

[12] Palm W, Webb E, Hernández-Quevedo C, et al. Gaps in coverage and access in the European Union. Health Policy. 2021;125(3):341–50. 10.1016/j.healthpol.2020.12.011. 33431257 10.1016/j.healthpol.2020.12.011

[13] Scholz N. Addressing health inequalities in the European Union: Concepts, action, state of play (2020) https://www.europarl.europa.eu/RegData/etudes/IDAN/2020/646182/EPRS_IDA(2020)646182_EN.pdf

[14] Goldsack JC, Coravos A, Bakker JP, et al. Verification, analytical validation, and clinical validation (V3): the foundation of determining fit-for-purpose for Biometric Monitoring Technologies (BioMeTs). NPJ Digital Med. 2020. 10.1038/s41746-020-0260-4. 10.1038/s41746-020-0260-4 PMC7156507 32337371

[15] TransCelerate Biopharma Inc. Sponsor experience implementing decentralized clinical trial (DCT) elements - DCT implementation survey results https://www.transceleratebiopharmainc.com/wp-content/uploads/2024/04/Sponsor-Experience-Implementing-Decentralized-Clinical-Trial-DCT-Elements-DCT-Implementation-Survey-Results_vFINAL-1.pdf

[16] Innovative Medicines Initiative (IMI). Project Factsheet: Center of excellence – remote decentralised clinical trials. Trials@Home. https://www.imi.europa.eu/projects-results/project-factsheets/trialshome

[17] https://myscrs.org/best-practices-and-recommendations/

[18] European Commission - Directorate-General for Health and Food Safety, Recommendation paper on decentralised elements in clinical trials. 14 December 2022, pp.17–34. Online: https://health.ec.europa.eu/system/files/2023-03/mp_decentralised-elements_clinical-trials_rec_en.pdf

[19] Radial Trial (2020) https://www.radial.eu/en

[20] Committee for Human Medicinal Products. ICH E6 (R3) Guideline on good clinical practice (GCP) (2023) https://www.ema.europa.eu/en/documents/scientific-guideline/draft-ich-e6-r3-guideline-good-clinical-practice-gcp-step-2b_en.pdf

